# Understanding the Role of Hemodynamics in the Initiation, Progression, Rupture, and Treatment Outcome of Cerebral Aneurysm from Medical Image-Based Computational Studies

**DOI:** 10.5402/2013/602707

**Published:** 2013-07-02

**Authors:** Marcelo A. Castro

**Affiliations:** Grupo de Investigación y Desarrollo en Bioingeniería, Facultad Regional Buenos Aires, Universidad Tecnológica Nacional, CONICET, Medrano 951, CP 1179, Buenos Aires, Argentina

## Abstract

About a decade ago, the first image-based computational hemodynamic studies of cerebral aneurysms were presented. Their potential for clinical applications was the result of a right combination of medical image processing, vascular reconstruction, and grid generation techniques used to reconstruct personalized domains for computational fluid and solid dynamics solvers and data analysis and visualization techniques. A considerable number of studies have captivated the attention of clinicians, neurosurgeons, and neuroradiologists, who realized the ability of those tools to help in understanding the role played by hemodynamics in the natural history and management of intracranial aneurysms. This paper intends to summarize the most relevant results in the field reported during the last years.

## 1. Introduction

Stroke is the leading cause of long-term disability and the third cause of death in the Western World. Subarachnoid hemorrhage (SAH) is one of the most severe types of stroke, which usually occurs when an intracranial aneurysm ruptures [1]. SAH refers to a leakage of blood into the subarachnoid spaces, which is a continuous space between the supratentorial and infratentorial compartments. A greater concentration of the blood products around the site of bleed is usual. When hemorrhage extends into the adjacent parenchymal structures and ventricular system, it results in a higher morbidity and mortality rate [2]. Vasospasm, in which blood vessels constrict to restrict blood flow, is a serious consequence of SAH because it may cause ischemic brain injury and permanent brain damage due to lack of oxygen in parts of the brain [3]. Understanding of the underlying mechanisms that result in injury after SAH is limited. However, a number of studies indicate that apoptosis may play a major role in the pathogenesis of secondary brain injury after SAH [4]. Elevated intracranial pressure is an important consequence of aneurysmal SAH that often results in not only decreased cerebral perfusion but also secondary clinical decline [5]. 

Intracranial aneurysms tend to initiate at or near arterial bifurcations, mostly in the circle of Willis. The yearly risk of subarachnoid hemorrhage for an unruptured intracranial aneurysm is approximately 1% for lesions 7 to 10 mm in diameter. The optimal management of unruptured aneurysms is controversial, and current decision making is mainly based on aneurysm size and location, as derived from the International Study of Unruptured Intracranial Aneurysms (ISUIA) [6]. Current guidelines suggest that, with rare exceptions, all symptomatic unruptured aneurysms should be treated. On the other hand, incidental aneurysms less than 5 mm in diameter should be managed conservatively in virtually all cases, while almost all larger aneurysms should be treated. Exceptions depend on patient age among other factors. The microsurgical clipping rather than endovascular coiling should be the first treatment choice in low-risk cases [7].

Cerebral arteries do not have an external elastic lamina, have sparse medial elastin, lack supporting perivascular tissue and structural irregularities at the apex of their bifurcations [8, 9]. These factors would indicate that cerebral arteries are susceptible to a local weakening under the persistent action of hemodynamic loads, particularly in hypertension [10]. Although it is widely accepted that hemodynamics, particularly the wall shear stress, plays an important role in the development, growth, and rupture of cerebral aneurysms [11], there is still no agreement about what hemodynamic characteristics trigger the biological mechanisms associated to those processes in individual cases. One theory proposes that low values of wall shear stress (WSS) produce flow stagnation near the aneurysm dome, resulting in the accumulation of red blood cells, platelets, and leukocytes responsible for endothelium damage. This mechanism would favor the infiltration of white blood cells and fibrin within the arterial wall causing, weakening and rupture as could be observed in pathological tests of cadaveric specimens of cerebral aneurysms [12]. Another theory proposes that damage to the endothelium, which would be responsible for triggering the remodeling and arterial degeneration and the consequent weakening of the wall, can be the result of high WSS [13]. In addition, external forces due to contact of the aneurysm with extravascular structures can contribute to rupture [14]. Previous patient-specific image-based computational hemodynamic studies showed that ruptured aneurysms tended to have small impaction zones, complex or unstable flow patterns, and high WSS peak values [15–18], while other authors found associations between low WSS and rupture [19, 20]. Similar discrepant results were found when aneurysm growth was analyzed using personalized models [21–23]. The wall shear stress plays an important role in the development and rupture of cerebral aneurysm. However, aneurysms rupture when wall tension exceeds the strength limit of the wall tissue. At this moment, the aneurysm wall mechanics is not completely understood and requires computational simulations able to consider the interaction between the wall and the fluid in a personalized manner, which needs prior knowledge of quantities difficult to be measured in vivo. Different strategies have been recently proposed to address this problem [24].

During the last years, a considerable number of image-based patient-specific computational fluid dynamics blood flow simulations in domains containing endovascular devices were presented. Computational algorithms devised for modeling the deployment of endovascular devices, such as coils or stents, have gained an increasing interest given their ability to predict hemodynamic changes and evaluate outcome [25]. Patient outcome depends on a number of processes that may take place on the deployment site as a result of the interaction between the device and both the blood flow and vessel wall.

One of the major problems with coiling is the recanalization, which can be caused by either coil compaction or aneurysm growth [26–28], both associated to the hemodynamic forces acting on coils and the aneurysm wall over time [29, 30]. A recent study indicates that 28.6% of aneurysms recurred within one year of coiling and that the recurrence rate increased in time [31]. Similar finding had been previously reported by another group, where 33.6% of aneurysms recurred within one year of coiling [32]. The most recent published data reveals that recurrence rate is even higher: 36.5% recurrence rate at 9 months [33]. Data from the International Subarachnoid Aneurysm Trial (ISAT) group indicates that the higher aneurysm rate of recurrence is associated with a higher rebleeding rate, given that the rebleeding rate of coiled aneurysms was eight times higher than that of surgically treated aneurysms [34].

An alternative option is the use of flow diverter devices, which is an emerging neurovascular technique based on self-expandable braided stent for treating intracranial aneurysms. Variability in outcome has underscored a need for investigating the hemodynamic effect of fully deployed stents in patient-specific aneurysms [35, 36]. Clinical experiences with stent-based flow diversion have been reported [37–39]. The influence of the stent design on the intra-aneurysmal hemodynamics patterns using computational simulations has been studied for some cases [35, 40]. Recently, the results of a trial for evaluating a pipeline embolization device (PED) for the intracranial treatment of aneurysms were presented. A total of thirty-one aneurysms were treated at four different centers using a PED, and all patients underwent clinical and angiographic evaluation that showed that angiographic occlusion was observed in the majority of cases [41]. A recent report showed cases where a stent used as a flow-diverting device to treat cerebral aneurysm removed a proximal stenosis resulting in an increase of the intra-aneurysmal pressure and a consequent bleeding [35]. Although unusual, cerebral aneurysms may coexist with a proximal artery stenosis. In that small percent of patients, such coexistence poses a challenge for interventional neuroradiologists and neurosurgeons to make the best treatment decision. According to previous studies, the incidence of cerebral aneurysms in patients with internal carotid artery stenosis is not greater than 5%, where the aneurysm is usually incidentally detected [42–44]. That percentage is less than 2% when the aneurysm was located in the same arterial circulation [45]. Alterations in intra-aneurysmal flows after plaque removal depend on a number of factors [42, 46, 47]. Some CFD studies have been performed to characterize those changes and their impact on aneurysm mechanisms [48, 49].

The development of computational methods for the study of blood flow in patient-specific domains reconstructed from medical images has advanced the understanding of the mechanisms of interaction between the blood flow and the arterial wall, the initiation and development of cerebrovascular diseases, and the flow alterations generated by endovascular devices, which helps elucidating the natural history of cerebral aneurysms and evaluating treatment options. The purpose of this review is to present results from numerical experiments performed by prestigious research teams that have made valuable contributions to the field during the last decade. The paper is organized as follows. The first section presents a series of studies where patient-specific computational fluid dynamic models of cerebral aneurysm were validated using both experimental and in vivo data and analytical solutions. The aim of the second section is to review sensitivity studies whose aim was to analyze the dependence of the numerical solutions on assumptions and parameters of computational models. The third section includes works where associations between hemodynamic characteristics and initiation, growth, and rupture were investigated. In the Following, the most relevant contributions to study aneurysms in the anterior communicating artery from image-based patient-specific models are discussed. That particular location is where about one-fourth of all cerebral aneurysms form. The next section focuses on the computational modeling of endovascular devices, such as coils and stents. One more section is devoted to present works where associations between hemodynamic patterns and thrombus formation were studied. Finally, a number of research papers, where wall dynamics is taken into account, are discussed. At the end, the author suggests some additional review articles about related topics, such as techniques for image-based of blood flow and vessel wall dynamics, biomechanical behavior of the healthy and diseased cardiovascular system, and connections between a variety of risk factors of intracranial aneurysms.

## 2. Validation of Patient-Specific CFD Aneurysm Models

The first image-based patient-specific methodology for blood flow computational fluid dynamic simulations under realistic flow conditions were presented in 1998 by Taylor et al. [50] and Milner et al. [51]. A few years later, sophisticated image processing and vascular reconstruction algorithms were incorporated to model complex geometries like the whole circle of Willis [52] and the posterior circulation with a giant intracranial aneurysm [53]. Although different approaches for each stage of the pipeline have been proposed, all those strategies rely on high quality and resolution angiography images, algorithms for image processing and reconstruction of the vascular boundary from segmented images, three-dimensional grid generation, numerical integration of the fluid dynamics and solid dynamics partial differential equations, and data analysis and visualization tools. CFD may supplant traditional engineering flow measurement techniques such as laser Doppler velocimeter (LDV) or particle imaging velocimeter (PIV). However, the clinical applicability of CFD-based methodologies required an extensive validation [54].

Sun et al. performed a phantom based experimental validation of their computational fluid dynamics methodology for cerebral aneurysms. Virtual angiograms were generated, and quantitative comparison to the experimentally acquired angiograms showed a good agreement [55]. Karmonik et al. qualitatively compared simulated intra-aneurysmal flow distributions with values acquired using two-dimensional phase-contrast magnetic resonance images (2dPC-MRI). Three patients were considered in that study. Inflow into the aneurysm was modeled on the basis of the volumetric inflow rates measured with 2dPC-MRI. Flow patterns measured with 2dPC-MRI showed flow characteristics that qualitatively reproduced the CFD simulations considering a Newtonian rheology and rigid walls, while differences in the calculated magnitude of the velocity were found. It was reported that 2dPC-MRI was unable to resolve small secondary flow patterns originating from the complex aneurysm geometry that can be appreciated in the CFD simulations. Additionally, multidirectional flow in aneurysms may introduce further artifacts in 2dPC-MRI images [56]. The same authors utilized that methodology to compare the velocity distribution within an anterior communicating artery aneurysm measured using 2dPC-MRI and computed by CFD simulations. Vascular model was limited to the A1 and A2 segments of the left and right anterior cerebral arteries (ACAs). Inflow conditions were based on flow measurements. Although some differences were reported, features in intra-aneurysmal cross section of AComA aneurysms calculated with CFD using patient-specific inflow boundary conditions were found to be in good agreement with features measured in vivo with 2dPC-MRI [57]. Castro et al. compared CFD steady and unsteady blood flow simulations in idealized models and established a relationship between the error and the number of elements in a cross-sectional area of the vessels for both Newtonian and Casson rheologies. Those estimations are useful for grid generation with element sizes automatically adjusted according to the vessel diameter in patient-specific models [58]. Cebral et al. compared hemodynamics in normal cerebral arteries from 4D phase-contrast magnetic resonance images (4dPC-MRI) and image-based CFD simulations. Three young normal subjects were scanned on a 3.0 Tesla scanner. Flow pattern visualizations showed that qualitatively the major flow structures like swirling flows and flow direction in communicating arteries observed in 4dPC-MRI and CFD simulations coincided. However, a number of differences were reported: (a) the velocity magnitudes tended to be higher in the CFD models, (b) secondary flows (rotational or nonaxial velocity components) were weaker in the 4dPC-MRI images, making the flow pattern more parallel or laminar, (c) flow recirculation regions observed in the CFD models near vessel bifurcations were not well captured by 4dPC-MRI images, and (d) the flow fields derived from the 4dPC-MRI images were not divergence-free, and some streamlines stop inside the vascular domain [59].

In another work, Cebral et al. presented a qualitative comparison between CFD simulations and cerebral angiography. Three patients with cerebral aneurysms and different flow types were selected for that study. Rotational angiography images were obtained along with biplane angiography. Vascular models were used for blood flow finite element simulations under pulsatile flow conditions. In addition, synthetic angiograms were produced for each patient, where the entire flow field previously computed was used to simulate the transport of a virtual dye by solving the transport equation. At the inlet of the models, a time-dependent concentration was prescribed mimicking the contrast injection used in the actual angiograms. It was reported that CFD modeling was in good agreement with imaging of the intra-aneurysmal flow structures by conventional angiography. According to the authors, minor differences were more prominent during the aneurysm washout, and some possible causes were rheology and inflow waveforms [60]. van Ooij et al. utilized a real-size aneurysm phantom to study complex flow patterns and compare PC-MRI imaging, PIV measurements, and CFD simulations. Good quantitative agreement was found between them. The differences between CFD simulations and PC-MRI had a root mean square error between 4% and 5%, while differences between CFD and PIV reached 12% [61]. Ford et al. compared CFD predictions against particle image velocimeter (PIV) measurements of flow dynamics in anatomically realistic aneurysm models. They demonstrated that CFD not only captures the gross flow patterns but also many of the finer flow details and their associated cycle-to-cycle variations [62]. All those observations suggested that CFD simulations are able to obtain more accurate flow estimation than in vivo techniques.

## 3. Sensitivity of CFD Simulations 

### 3.1. Reconstruction

The previous section showed a series of works that claim that computer simulations exceed the predictive ability of in vivo measurements. However, personalized blood flow simulations depend on a number of assumptions whose impact on the results had to be carefully analyzed before pursuing clinical applicability. The first sensitivity study designed to determine the influence of computational model assumptions and parameters on a number of intra-aneurysmal flow characteristics whose association with biomechanical processes was investigated was performed and presented by Cebral et al. in 2005 [63]. Four vascular models were reconstructed from three-dimensional rotational angiography (3DRA) images, which is the preferred modality to diagnose and follow up cerebral aneurysms due to its high resolution and contrast. Flow characteristics under study included flow complexity, flow stability, impingement region, impingement size, and WSS distributions. Assumptions and parameters considered in that study included mean inflow rate, distal flow division, blood rheology, reconstruction of small vessels, and reconstruction method. It was found that that all characteristics remained unchanged when variations in model parameters were considered. However, in one case that whose reconstruction required manual editing, differences in the geometry resulted in changes in the flow characterization. Geers et al. [64] investigated the differences in hemodynamics characteristics when comparing simulations using ten cerebrovascular models harboring aneurysms reconstructed from both 3DRA- and CTA-based images. Although relatively large differences were found for all evaluated quantitative hemodynamic variables among subjects, the main flow characteristics were reproduced across imaging modalities. It was reported that the lower spatial resolution of CTA made it difficult for the segmentation algorithm to distinguish vascular structures that were in close proximity to each other. That result was later corroborated by Augsburger et al. [65], who studied the effects of segmentation on cerebral aneurysm's morphological parameters and on blood flow patterns computed using computational fluid dynamics. All three teams concluded that geometry is critical in patient-specific blood flow simulations and most efforts would be directed towards automated and accurate reconstructions.

### 3.2. Boundary Conditions and Heart Rate

Jiang and Strother [66] studied the influence of heart rate (60, 100, and 150 bpm) in the intra-aneurysmal hemodynamics in two patient-specific models with special attention to any flow disturbance that might occur at or near the aneurysm as the heart rate increased. The authors reported an increment in flow complexity and WSS values at higher rates. Marzo et al. [67] investigated the variability of certain hemodynamic parameters with boundary conditions. In that preliminary study-differences were found between results obtained with patient-specific and modeled boundary conditions, which were attributed to underlying differences in the Reynolds number of the flow approaching the aneurysms. In fact, discrepancies were significantly reduced when considering normalized indices, suggesting a certain degree of linearity in the results and the important role played by geometry in intra-aneurysmal hemodynamics. The results presented in that work showed that modeled boundary conditions allow realistic predictions of intracranial aneurysm hemodynamics and offer a viable means for finding correlations with rupture in large cohort studies. Different conclusions were reported by Karmonik et al. [68] when they studied the dependence of WSS calculations on the waveform imposed at the inlets of the vascular models. For each one of the six patient-specific models, two different blood flow simulations were performed. The first one considered an average waveform from normal subjects. The second one used the patient's waveform, which was acquired using 2D phase-contrast magnetic resonance imaging. Large differences in WSS and oscillatory shear index were reported. Comparability of CFD simulations when non patient-specific wave forms are imposed to the inlets of the vascular models requires flow rate normalization [16, 58].

### 3.3. Rheology

Fisher and Rossmann [69] created four idealized models with different morphological characteristics to study the effect of blood rheology on aneurysm hemodynamics. The blood's non-Newtonian behavior was found to have a more significant effect on the fluid forces within aneurysms than in the parent vessels and to be more important during diastole. It was also found that non-Newtonian behavior was also more influential in aneurysms at bifurcations than for sidewall aneurysms in either straight or curved vessels. Similarly, Castro et al. [58] compared CFD steady and unsteady blood flow simulations in idealized models and established the maximum difference between Newtonian and Casson profiles in unsteady blood flow simulations, which occurred in the diastolic part of the cardiac cycle. Jou and Mawad [70] found that Newtonian flow overestimates the impingement size in a giant internal carotid artery aneurysm, which may affect predictions based on that parameter. Khanafer et al. [71] showed that non-Newtonian wall shear stress is greater during the peak systole in a limited number of aortic aneurysm models. Rayz et al. [72] found no significant differences between low wall shear stress regions that may be associated with risk of thrombus formation using Newtonian and non-Newtonian computational fluid dynamic simulations in three patient models. However, accounting for non-Newtonian behavior improved the agreement with observations using longitudinal MRI studies. Xiang et al. [73] showed that Newtonian viscosity model could overestimate normalized wall shear stress in three internal carotid artery saccular aneurysms. Castro et al. [74] performed a similar study over five patient-specific aneurysm models and found no clear correlation between low WSS regions and regions where any of the rheologies predict larger WSS values. A nonnegligible change in WSS vector orientation as large as 20° in regions of low WSS was observed. The location of those regions was not necessarily associated with regions where large differences in WSS occur when considering different rheologies. These observations may indicate that the realistic geometry plays an important role in the intra-aneurysmal hemodynamic characteristics, resulting in low flow regions where either the Newtonian or non-Newtonian WSS may be larger than the other one, exhibiting also differences in the vector orientation. Valencia et al. [75] investigated the effect of non-Newtonian rheology on the flow pattern of typical aneurysms at the basilar artery and found that the non-Newtonian fluid assumption yields more stable flows than a Newtonian fluid, for the same inlet flow rate. Effects of rheology on velocity field and WSS were also investigated in 20 patient-specific lateral aneurysm models. Linear correlations between the WSS on the aneurysm fundus at peak systole for lateral aneurysms with an area index were found [76].

### 3.4. Wall Motion

Although most previous CFD analysis considered rigid walls, that assumption may affect estimation of hemodynamic quantities. Many of the quantitative results of pulsation measurement reported in the literature correspond to experiments with phantoms, simulated images, or experimental models [77–80]. A Methodology to estimate wall motion from X-ray dynamic imaging and impose the time-dependent deformation on the vascular CFD models reconstructed from 3DRA images was presented [81]. The blood flow characterization obtained from numerical integration of the Navier-Stokes equations in a rigid wall model did not significantly differ from that in compliant models where the deformation field was extrapolated from the bidimensional measurements [58, 81]. An improved approach was also presented using digital subtraction angiography images (DSA) and B-splines free form deformations to quantify aneurysm wall motion. Statistically significant differences in pulsation were found [82]. In a recent work, a methodology to both estimate regions of high wall motion and reconstruct CFD vascular models from 4D computerized tomographic angiography (CTA) data sets was presented. A connection between regions of high pulsation amplitude and regions of high wall shear stress was suggested [83]. Additionally, a similar strategy was used to quantify vascular wall displacement from cardiac-gated 4D CTA at different locations [84]. That study included nineteen aneurysms from fourteen patients. Pulsation of the aneurysm and its surrounding vasculature during the cardiac cycle could be assessed from ECG-gated CTA data. The percentage of aneurysmal volume change ranged from 3% to 18%. However, the study showed that the amount of volume change estimated by the method is not related to aneurysm size. Other authors presented methodologies to simulate the fluid-structure interaction between the blood and the vessel wall [85, 86]. Those and other works are discussed under the section “Wall Biomechanics”.

## 4. Associations between Hemodynamic Features and Aneurysm Initiation, Growth, and Rupture

Intracranial aneurysms preferentially localize close to bifurcations and curvatures where hemodynamics are complex. While extensive knowledge about low WSS has been generated in the past based on its strong relevance to atherogenesis, high WSS has emerged as a key regulator of vascular biology and pathology as well, receiving renewed interests [86]. Chronic high WSS not only stimulates adaptive outward remodeling but also contributes to saccular intracranial aneurysm formation at bifurcation apices or outer curves and atherosclerotic plaque destabilization in stenosed vessels. As an adaptive response to chronically elevated high flow, an artery undergoes expansive or outward remodeling to restore WSS back to baseline levels [87–89]. On the other hand, low values of WSS may produce flow stagnation near the dome of the aneurysm, resulting in the accumulation of red blood cells, platelets, and leukocytes responsible for endothelium damage. This mechanism would favor the infiltration of white blood cells and fibrin within the arterial wall causing, weakening and rupture as could be observed in pathological tests of cadaveric specimens of cerebral aneurysms [12].

### 4.1. Aneurysm Initiation

Alnæs et al. [90] studied the effect of asymmetries in branch angles and differences in vessel radii on WSS and pressure distributions in CFD models of the Circle of Willis over a ten-patient population. Numerical simulations showed high WSS at locations where aneurysms are frequent and in anatomic variants known to be associated with an increased risk for aneurysm development. Mantha et al. [91] studied the hemodynamics patterns of three image-based paraclinoid aneurysm models. Simulations revealed an area of relatively low and rotating WSS at the location at which each aneurysm had developed. Castro et al. [22] studied three AComA aneurysm models and found that the aneurysm always formed in a region of high or moderate WSS, but not in a region under low WSS. Normal models were created from pathological ones by applying a Laplacian filter. In another study, Kulcsár et al. [23] presented a relationship between rupture and coexistence of high WSS and high positive spatial WSS gradient observed in three patients scanned before and after aneurysm formation. Ford et al. [92] presented a novel methodology to automatically remove saccular aneurysms from vascular models using a Voronoi based approach. The evaluation of the methodology was performed over patients with aneurysms at different locations. The CFD analysis was performed over five selected cases. From those simulations, it was observed that the gradient oscillatory number (GON) may be a sensitive hemodynamic marker for aneurysm formation, since GON showed modest elevated values near aneurysm location in four of five cases. WSS distributions demonstrated no consistent hemodynamic feature (e.g., low shear) associated with the nominal site of aneurysm formation.

### 4.2. Aneurysm Growth

Boussel et al. [93] performed a longitudinal study over seven patients to investigate possible associations between regions of low WSS and regions where the aneurysm grew. A relatively short portion of the parent vessel was considered according to the reported data. Manual coregistration was performed in order to achieve alignment. Data analysis indicated that aneurysms tended to grow in regions of low average WSS. Sugiyama et al. [94] studied two posterior inferior cerebellar artery aneurysms. They found different hemodynamic characteristics and growing patterns: a proximal multilobular aneurysm was growing a bleb near the inflow zone with high local velocity and physiological levels of WSS, whereas a distal rounded aneurysm was growing the entire sac featuring low and unstable intra-aneurysmal flow, with low WSS and higher OSI. Thus, the authors concluded that growing aneurysms may have heterogeneous hemodynamic, morphologic, and vascular characteristics associated with different mechanistic pathways. A patient-specific CFD study over three cerebral aneurysms that enlarged with blister formation during a follow-up period of about ten months showed that blister formation occurs in low WSS regions [21]. Another study over thirty well-defined blebs in twenty intracranial aneurysms showed that eighty percent of blebs originated in regions of high WSS [18]. Recently, independent works showed that aneurysms tend to initiate in regions of moderate and elevated WSS [22] and regions of high WSS or high WSS spatial gradient [23]. Valencia et al. [95] investigated the relationship between WSS and aneurysm area index, which is the ratio between the aneurysm area and the artery area at the model inlet. They found a correlation between those quantities in a study that included 34 patient-specific CFD models reconstructed from rotational angiography images. Sforza et al. [96] studied the effect of perianeurysmal environment during the growth of a cerebral aneurysm. A selected case of a 19 mm basilar aneurysm immediately distal to the vertebrobasilar junction and in contact with the clivus bone was considered. The aneurysm was followed noninvasively with CTA imaging at 1-year intervals for a total of four years. The aneurysm was observed to grow against the bone resulting in a geometric change of the proximal artery and consequently in the aneurysm hemodynamics. A region of elevated WSS was observed to shift locations in time. The authors concluded that contact with perianeurysmal structures should be considered and analyzed to assess whether they could exert a significant influence on the geometric evolution and hemodynamic patterns of the intracranial aneurysms.

### 4.3. Aneurysm Rupture

Xu et al. [97] studied eight mirror aneurysms (ruptured and unruptured) located at the posterior communicating artery and found a correlation between low WSS and rupture. The wall shear stress, which had been time-averaged, was normalized by the WSS at the parent artery. Similar normalization had been previously used to analyze twenty middle cerebral artery aneurysms and also found high WSS values in the group of ruptured aneurysms accompanied with low WSS in their domes, which would suggest that low WSS values may be responsible for aneurysm rupture [19]. In that study, CFD models were truncated close to the aneurysm neck resulting in simplified flows lacking of secondary structures. The effect of parent artery on intra-aneurysmal hemodynamics was later studied and higher WSS values in the aneurysm domes were found to be systematically related to those secondary flows [98, 99]. A different approach proposed by Castro et al. relied on the normalization of flow rate curves imposed at the inflows of the vascular models. That approach was utilized to study twenty six anterior communicating artery aneurysms. The maximum value of the WSS at the systolic peak in a ruptured group was in average more than twice that in the unruptured group [16]. A similar trend was found in a cohort of forty-one terminal aneurysms. Higher WSS values occurred close to the neck where the inflow jet splits [17]. A recent study by Miura et al. [100] analyzed 106 patients with aneurysms in the middle cerebral artery. The cohort was composed by 43 ruptured aneurysms and 63 unruptured ones. The authors could not find any significant difference in gradient oscillatory number and dome size between those groups, but they reported that lower WSS was significantly associated to aneurysm rupture. Although the authors mentioned that typical waveforms of the internal carotid artery were used, it is not reported whether or not those waveforms were scaled according the arterial cross-sectional area. Normalized WSS (NWSS) was also analyzed, and the authors reported that lower NWSS significantly correlates with rupture, but WSS was considered because it had the smallest *P* value. Previous patient-specific image-based computational hemodynamic studies showed that ruptured aneurysms tended to have small impaction zones, concentrated jets, and complex flow patterns [15]. The same trend was corroborated by Cebral et al. [18] in a study that included 210 cerebral aneurysms at different vascular locations. Flow division and jet concentration were found to be associated to high WSS values and rupture rate [15, 16]. Patti et al. [101] studied 41 carotid artery aneurysms and found that the pulsatility index (PI) was significantly higher (*P* < 0.001) at ruptured (1.93) than unruptured (0.59) aneurysm necks. Unruptured aneurysms tended to exhibit PIs close to that in the parent artery (0.61). Chien et al. [102] investigated small aneurysms in the carotid artery-ophthalmic artery. Most ruptured aneurysms had complicated flow patterns in the aneurysm domes, but all of the unruptured cases showed a simple vortex. Inside the aneurysms, the highest flow velocities were found either at the apex or neck. Higher and more inhomogeneous WSS distributions within ruptured aneurysms were observed in comparison with the unruptured ones. 

### 4.4. Low WSS—High WSS

Regarding low WSS and high WSS as being responsible for triggering aneurysm mechanisms, as shown before, similar studies lead to different results and conclusions. Cebral and Meng [103] and Meng et al. [104] proposed that the high versus low wall shear stress controversy may be a manifestation of the complexity of aneurysm pathophysiology. Low WSS and high oscillatory shear index trigger an inflammatory-cell-mediated pathway, which could be associated with the growth and rupture of large, atherosclerotic aneurysm phenotypes, while high WSS, combined with a positive wall shear stress gradient, triggers a mural-cell-mediated pathway, which could be associated with the growth and rupture of small or secondary bleb aneurysm phenotypes [104].

## 5. Anterior Communicating Artery Aneurysm Models

 The anterior communicating artery (AComA) is a recognized site of aneurysm predilection accounting for nearly twenty-five percent of all cerebral aneurysms in several large studies [105, 106]. Due to the complexity and diversity of geometry and flow conditions in the AComA, it is not surprising that aneurysms of the AComA are considered the most complex of the anterior circulation [107]. Aneurysms in the AComA are more likely to have asymmetric A1 segments [108], furthermore to have exclusive filling angiographically from one A1 segment in up to 78% of cases [109]. Tarulli and Fox [110] performed a radiological examination and found that A1 dominance configuration is strongly associated with the presence of AComA aneurysms for 105 patients with intracranial aneurysms at that location. Hassan et al. [111] performed a sensitivity study using idealized computational models of the anterior communicating artery and found that aneurysms located in the AComA with differences of 50% or more between the two A1s areas are subjected to more flow stresses. Experimentally, AComA aneurysms can be produced in hypertensive rats by unilateral ligation of the common carotid artery [112], further supporting a causative relationship between increased flow and aneurysm formation. A computational study found a possible correlation between AComA aneurysm formation, growth, and rupture with A1 dysplasia or hypoplasia. Increased WSS was found in the bifurcation when the diameter of the nondominant A1 segment decreased [113]. Previous in vitro modeling provided evidence of this effect. Using a silicone replica of a lethal anterior communicating artery aneurysm and imaging of fluid slipstreams, it was observed that with symmetrical flow conditions slipstreams rarely enter the aneurysm, but with asymmetric alterations in flow complicated flow patterns were identified within the aneurysm [114].

Three-dimensional rotational angiography (3DRA) is the preferred modality to diagnose and support treatment of cerebral aneurysms. However, in order to properly image the AComA, two 3DRA images are required. From the vascular reconstruction point of view, two independent computational models must be generated from the images acquired during the contrast injection at the left and right internal carotid arteries, and a final model is reconstructed by fusion. A methodology to overcome that limitation was designed and utilized to investigate the hemodynamics at the AComA and possible associations with aneurysm initiation and rupture [115]. A first observation is that AComA aneurysm flows dominated by only one A1 segment of the anterior cerebral arteries (unilateral) have a flow characterization independent of the mean flow rates imposed at the feeding arteries, the waveform, and phase shifts between those waves. On the other hand, those AComA aneurysm flows dominated by both A1 segments may experiment changes in the flow characterization. The main effect of changing the mean flow balance between the feeding arteries is an intensification of the flow impaction zone, resulting in increased magnitude of the WSS for the inflow jet with increased flow. The magnitude of the peak in WSS depends on whether the inflow jets interfere constructively or destructively. Constructive interference may lead to a peak WSS in excess of the base case of balanced flow. In contrast, changing the relative phase or the shape of the inflow waveforms has a more dramatic effect. Those changes introduce extratemporal dependencies of the velocity field that make the regions of elevated WSS travel along the surface of the aneurysm during the cardiac cycle. In the absence of aneurysms, phase or waveform shape differences in the inflows would induce to-and-fro motions of blood in the AComA [116]. Furthermore, Karmonik et al. [117] showed that hemodynamic patterns changed when flow rates at the A1 segments are altered with respect to the baseline defined by PC-MRI flow measurements in the same patient, but keeping the total flow rate unchanged. The maximum change observed in the aneurysm average WSS when increasing the right AComA flow rate to 68% was as high as 43%. According to the data presented in that paper, the model only included the A1 and A2 segments of the ACA; therefore, it is not clear if those differences would be so relevant when normalized flow rates are imposed at the ICAs. However, it is clear that using patient-specific inflow conditions would help in minimizing the effects of model assumptions.

Castro et al. [16] performed a patient population study that included twenty-six computational hemodynamics models of AComA aneurysms, which showed interesting associations between flow patterns and aneurysm rupture. The cohort was composed of 16 patients with bilateral A1 segments (58%) and 11 patients with one A1 segment missing (42%). Similar percentages were found in a previous study where 45% of the patients with AComA aneurysms showed a hypoplastic A1 segment [109]. In the paper mentioned previously, it was reported that exclusive filling of the AComA aneurysm from one of the A1 segments had been observed in 75% of the bilateral group. That was in good agreement with 78% of the cases reported in a previous study [110]. Four different flow types were proposed based on how the main jet split in different number of subjets when reaching the aneurysm. The two groups accounting for the highest number of ruptured aneurysms had a main jet entering directly the aneurysm and impacting the dome before being redirected towards the A2 segment of the anterior cerebral arteries (type D) and a main jet impacting the neck of the aneurysm (type C). In this last case, one of the subjets enters the aneurysm while the other one redirects towards one of the A2 segments. This configuration is responsible for the highest WSS values at the systolic peak and the highest risk of rupture among all groups (87%). Additionally, it was found that the peak WSS averaged over the ruptured groups (271 dyn/cm^2^) doubled that over the unruptured group (114 dyn/cm^2^). These findings may indicate that high WSS was responsible for aneurysm rupture. Another study considered three AComA aneurysm models and investigated the hemodynamic differences between the original model and the model before the aneurysm initiated. Laplacian filters were applied in order to remove the aneurysm. It was found that the aneurysm always formed in a region of high or moderate WSS, but not in a region under low WSS [22]. Recently, Hodis et al. [118] performed a CFD analysis of the hemodynamics of an AComA that spontaneously ruptured immediately following three-dimensional rotational angiography. Subsequent digital subtraction angiography allowed for the localization of the point of rupture within the aneurysm dome. CFD analysis demonstrated a concentrated jet that impinged directly at the site of rupture. The authors reported that peak systolic pressure and WSS were both maximal near the rupture location.

## 6. Computational Modeling of Blood Flow around and past Endovascular Devices

 The effectiveness evaluation of methods using endovascular devices such as stents or coils to treat cerebral aneurysms in a patient-specific and device-specific basis has become a major challenge for computational scientists and engineering researchers during the last years. The success of endovascular interventions depends on a number of processes that take place on the deployment site as a result of the interaction between the device, the blood flow, and the vessel wall. Coil embolization therapy has been subject to criticism for a consistent failure to achieve the same level of durable and complete aneurysm exclusion that surgical aneurysm clipping typically provides [41]. Regarding stents, they have been extensively used as flow diverter devices, which is an emerging neurovascular technique based on self-expandable braided stent for treating intracranial aneurysms. Variability in outcome has underscored a need for investigating the hemodynamic effect of fully deployed stents in patient-specific aneurysms [35, 36]. In a recent study of experimentally created aneurysms in twenty-one canines, Darsaut et al. [119] reported that flow diverters may succeed in treating straight sidewall aneurysms, but the same device repeatedly fails to occlude curved sidewall and end-wall bifurcation aneurysms.

In 2004, Stuhne and Steinman presented a body-conforming methodology to model blood flow past a flow diverter stent in an idealized aneurysm model [120]. A different strategy was proposed by Cebral and Löhner [121] in 2005. The approach presented there consisted in embedding the devices within the domain instead of generating a body-conforming grid, which is extremely difficult and tedious. The embedded methodology presented there was reported to be simple and fast and to fit very naturally in the context of endovascular device simulation. The main limitation of that methodology is the low resolution close to the device surface. The authors showed that that problem can be minimized by using adaptive grid refinement techniques to increase resolution around the embedded devices. Virtual angiograms were generated for different coil packing in a patient-specific vascular model harboring a terminal aneurysm to evaluate the impact of the device on the aneurysm, where the blood flows slowly with increasing coil packing. The same methodology was utilized for a stent as a flow diverter in both idealized and patient-specific model of a lateral aneurysm.

One of the major problems with coiling is the recanalization, which can be caused by either coil compaction or aneurysm growth [26–28], both associated to the hemodynamic forces acting on coils and the aneurysm wall over time [29, 30]. Low rate of complete occlusion is mostly observed in large and wide-neck aneurysms [32, 122]. Very large and giant aneurysms usually require a series of retreatments [123]. The failure of endosaccular techniques to achieve a complete and durable occlusion of aneurysms has been associated to limitations with respect to the volumetric packing of the aneurysm sac with coils, inherent difficulties associated with achieving a continuous reconstruction of complex aneurysm neck defects, and failure of the strategy to address the underlying diseased parent vessel [124]. Morales et al. [125] studied the dependence of coil packing density on aneurysm hemodynamics. They reconstructed three patient-specific vascular models, and blood flow simulations were carried out before virtual treatment after placing coils using three different coil configurations and packing density. They reported that the flow velocity was reduced more than 50% when packing density was about 12%. However, a high dependence on the coil configuration was observed. For higher densities (>30%) they observed a damping effect that resulted in a stable hemodynamic condition inside the aneurysm.

Preliminary clinical experiences with stent-based flow diversion have been reported [37–39]. Recently, the results of a trial for evaluating a pipeline embolization device (PED) for the intracranial treatment of aneurysms were presented. A total of thirty-one aneurysms were treated at four different centers using a PED, and all patients underwent clinical and angiographical evaluation one month and six months after treatment. Complete angiographic occlusion was observed in the majority of cases (>90%) [41]. Cebral and Löhner presented and evaluated a novel methodology to efficiently embed endovascular devices in patient-specific models [121] and utilized it to analyze the impact of the conformability characteristics of the stents on potentially occluded side branches, which may lead to stroke [126]. In 2008, the results of the virtual stenting challenge (VISC) were presented. The aim of that work was to establish the reproducibility of state-of-the-art hemodynamic simulation techniques in patient-specific stented and unstented models of intracranial aneurysms [36]. Six different institutions utilizing their own methodologies participated in that study. In both stented and unstented models, comparable results across different techniques and teams were achieved. Although differences in the magnitude of WSS and velocity were observed, WSS and velocity distribution and aneurysmal flow activity were reproducible across participant groups for both stented and unstented simulations. Ma et al. [127] presented and tested a finite element analysis based methodology of simulating mechanical deployment of flow diverters on a patient-specific and device-specific basis. The three-dimensional finite beam element models accounting for interactions between stent strands and between stent and other deployment components captured the mechanical responses of braided stents including the foreshortening effect of flow diverters. The influence of the stent design on the intra-aneurysmal hemodynamics patterns has been studied for some cases [40].

Larrabide et al. [128] reconstructed twenty-three vascular models 3DRA images of patients with ICA aneurysms to study WSS, velocity, residence time, turnover time, and intra-aneurysmal pressure using patient-specific blood flow simulations before and after virtual placement of a flow diverter. They observed that all hemodynamic variables exhibited significant reductions inside the aneurysm but the mean intra-aneurysmal pressure value, although the maximum pressure was decreased and the minimum pressure was increased. However, a recent report showed three cases where a stent used as flow-diverting devices to treat cerebral aneurysm removed a proximal stenosis, which resulted in aneurysm bleeding and patient death [35]. Computational simulations showed that in all those cases endovascular interventions resulted in an increase of the intra-aneurysmal pressure.

 Although unusual, cerebral aneurysms may coexist with a proximal artery stenosis. In that small percentage of patients, such coexistence poses a challenge for interventional neuroradiologists and neurosurgeons to make the best treatment decision. According to previous studies, the incidence of cerebral aneurysms in patients with internal carotid artery stenosis is not greater than 4.9%, where the aneurysm is usually incidentally detected [42–44]. Another retrospective study of 853 patients with stenosis in the carotid bifurcation showed that 46 patients (5.4%) had a cerebral aneurysm. That percentage was less than 2% when the aneurysm was located in the same arterial circulation [45]. Other studies have reported similar frequencies ranging between 1% and 3% [129–131]. A case report showed that a patient with a stenosis in the carotid artery and an asymptomatic unruptured aneurysm (5.0 × 10.0 mm) in the same artery at the posterior communicating artery bifurcation underwent a successful endarterectomy. The aneurysm would have been treated a few months later; however, the patient died after a subarachnoid hemorrhage five months after the endarterectomy, but before the aneurysm treatment. Autopsy revealed that the aneurysm had grown up to 14.0 × 10.0 mm before bleeding [46]. Another case report describes the treatment option for a patient with similar pathological characteristics. A stenosis in the carotid artery was located proximal to an unruptured aneurysm (14.0 × 8.0 mm) at the ophthalmic artery bifurcation in the same vascular circulation. A stent was successfully deployed to remove the stenosis. A few months after the intervention, no change was observed in the aneurysm size and the aneurysm was successfully treated with a coil embolization procedure [42]. Simultaneous interventions were also reported [47]. For low and mild stenoses, flow alterations in the aneurysm sacs are limited when the aneurysm is located far downstream in the same circulation. However, for distal aneurysms close to the stenosis, intra-aneurysmal hemodynamics may be significantly affected by the stenosis. According to a previous study, all patients with severe stenoses (stenosis area reduction greater than 84%) exhibited a flow reduction greater than 30% when compared to the contralateral circulation [132]. Consequently, the intra-aneurysmal hemodynamics may significantly change depending on how the ipsilateral flow rate increases after intervention. The management of carotid artery stenosis is well established for symptomatic stenosis area reduction indices above 69%, but the optimal approach for managing lower degrees of narrowing remains uncertain [133]. When a low-degree carotid artery stenosis coexists with a distal cerebral aneurysm, flow alterations in the aneurysm sacs are limited when the aneurysm is located far downstream in the same circulation [48]. However, it was reported that for distal aneurysms close to a mild stenosis, intra-aneurysmal hemodynamics may be significantly affected by the stenosis. That computational study also showed significant differences in blood flow and WSS depending on the distance between the aneurysm and the stenosis and their relative position [49]. 

## 7. Blood Flow and Thrombus Formation

The formation of intraluminal thrombus is considered as one of the most severe complications during the treatment of cerebral aneurysms [134]. Thrombus formation in intracranial aneurysm, while sometimes it stabilizes lesion growth, can present an additional risk of thromboembolism [135]. The presence of intraluminal thrombus within the cerebral aneurysm usually may lead to the distention of the wall and to increase the occurrence of neurological symptoms [72]. Cases of complete spontaneous thrombosis in the unruptured cerebral aneurysm have been discovered in recent years due to the advancement of neuroradiology [136]. However, reports on the correlations between the formation of intraluminal thrombus and the flow pattern, WSS distribution of the cerebral aneurysm as well as wall compliance are still limited. Rayz et al. [72] used rigid wall models to predict regions prone to thrombus formation. In a later study, Rayz et al. [135] analyzed three patients with thrombus-free intracranial aneurysm who proceeded to develop intraluminal thrombus detected in follow-up MR studies. Patient-specific CFD simulations predicted either an increased residence time or a low WSS at the locations where thrombus formed. Wang and Li [137] studied thrombus formation in patient-specific models using a fluid-structure interaction approach. The aneurysm wall was considered isotropic and linear elastic. Simulation result showed that thrombus-occupied areas were all located in the aneurysm bulges, which were the same as the regions where slow rotating vortices were formed. Low WSS values were also detected at those locations. It was shown that the maximum WSS predicted by the simulations was lower than the previous studies where the cerebral aneurysm wall was considered rigid. The authors explained that the flexibility of the wall enabled the aneurysm cavity to dilate, and hence, the direct impact coming from the pulsatile blood flow acting on the wall was weakened, thus resulting in lower WSS. A previous study suggested that excessively low WSS (lower than 1.5 Pa s) may facilitate the process of endothelial cell degeneration, while the arterial structure can maintain its normal physiological function when the magnitude of WSS is above 2 Pa s [138]. Xiang et al. [73] analyzed three patient-specific models with rigid walls and found that non-Newtonian rheology overestimates wall shear stress in intracranial aneurysm domes, and therefore underestimate the risk of thrombus formation.

## 8. Wall Biomechanics

 The wall shear stress plays an important role in the development and rupture of cerebral aneurysm. However, aneurysms rupture when wall tension exceeds the wall strength. Wall strength depends on local protease activity and rates of collagen synthesis, which are controlled by cellular responses to local hemodynamic loads. The arterial wall, which is a complex composite of structural proteins (mostly elastin and fibrillar collagen), resident cells, and a ground substance matrix, exhibit-viscoelastic characteristics. However, the assumption of hyperelasticity is sufficient in most physiologic and pathophysiologic cases [24]. At this moment, the aneurysm wall mechanics is not completely understood. Computational simulations that consider the fluid-structure interaction require prior knowledge of quantities difficult to be measured in vivo. In a recent experimental study, sixteen aneurysms underwent mechanical uniaxial stress under physiological conditions, temperature, and saline isotonic solution. A hyperelastic model was fitted to stress-strain curves, and each aneurysm was classified according to its biomechanical properties and rupture status. All unruptured aneurysms presented a more rigid tissue. However, wall thickness did not correlate to rupture status [139]. A further fluid-structure interaction computational study, where the aneurysm wall was considered as isotropic and homogeneous based on ex vivo measurements, showed significant differences between the displacements and volume variations corresponding to the soft and stiff tissue. Therefore, the detection of pulsation may provide further information to determine the risk of rupture [140]. In a previous study, wall tension was determined in a single computational model that considered the aneurysm wall as an isotropic nonlinear material. The authors reported that regions where wall tension reached high values appeared where aneurysms are more prone to rupture [141]. In a similar study, the wall tension was computed in a patient-specific hemodynamic model of a middle cerebral aneurysm using a fluid structure interaction strategy [141]. The arterial and aneurismal wall was treated as an isotropic nonlinear hyperelastic solid, and the aneurysm wall thickness was uniform. Maximum displacement was observed at the aneurysm dome at the systolic peak and was three times the aneurysm wall thickness. The wall tension was unevenly distributed. Other authors used Young's modulus and wall thickness from the literature to be imposed in the numerical simulations. Distal vessels are stiffer and thinner than the proximal vessels. In the aneurysmal region, the thrombus side was assigned to have a greater Young's modulus and thickness [142]. Balocco et al. considered the aneurysm wall as an incompressible linear elastic isotropic material, which does not capture the nonlinear anisotropic and layer-dependent nature of the stress-strain curves at high strains given by some hyperelastic constitutive models of the arterial wall. However, in that study the wall parameters were computed by means of an inverse problem using wall motion estimation from registered images at different times [143]. The parametric biomechanical model, given the initial aneurysm morphology (diastole), the patient blood flow, and an initial set of parameters describing the regional distribution of the wall elasticity, enabled the computation of the modeled aneurysm morphology (systole). The distance between this modeled morphology and the one obtained from the aneurysm pulsation was iteratively minimized to estimate the optimal set of elasticity parameters. According to in silico experiments performed in that study, the minimal spatial resolution needed to extract wall pulsation measurements with enough accuracy was of 0.1 mm. However, current routine imaging modalities do not have such a high spatial resolution, and therefore, the proposed data assimilation framework cannot currently be used on in vivo data to reliably estimate regional properties in cerebral aneurysms. The authors reported that the incorporation of fluid-structure interaction in a biomechanical model with linear and isotropic material properties did not have a substantial influence on the final results. In a recent study, the region of the aneurysm wall exhibiting high pulsation amplitude estimated from high time resolution dynamic tomographic angiography images correlated to the region of high wall shear stress computed in a patient-specific model using personalized inflow conditions and rigid walls [85]. Bazilevs et al. proposed an approach to compute wall shear stress and wall tension in four patient-specific vascular models using varying wall thickness [144]. It was assumed that the wall thickness at the inlet and outlet branches is 20% of their effective radii, which is defined as the radius of the circle that has the same area as a given inlet or outlet. The wall thickness for the remainder of the model was constructed by performing a smooth Laplace operator-based extension of the inlet and outlet thickness data into the domain interior. While the branch vessel wall thickness was accurately represented, the aneurysm dome thickness was overestimated. However, the authors reported that the resultant dome and branch vessel thickness fell well within the range of values reported for cerebral aneurysms. Valencia et al. [145] performed a computational solid dynamic (CSD), CFD, and FSI analysis of a patient-specific vascular model harboring two aneurysms. Wall thickness was assumed uniform, and a non-Newtonian rheology was used. The FSI results showed maximum WSS values of 8 Pa at the aneurysm domes and maximum displacements of 1.4 mm. with recirculation vortex in the region of low WSS, which is in agreement with previous reports [146]. However, vortex structure, WSS, effective stress, strain, and displacement of the aneurysm walls showed differences, depending on the type of modeling used. It is worth mentioning that accurately measuring wall thickness is still a challenge. Overestimation of wall thickness in black blood MRI imaging when compared to specimen-based thickness measurements has been reported [147, 148].

 A few years ago, Humphrey and Taylor [149] suggested that a new class of coupled computational tools for studying evolving vascular changes was needed. That new class should not only take into account the computational fluid dynamics and fluid-structure interaction, but also include long-term growth and remodeling models of the evolving wall, which were called fluid-solid growth (FSG) models. The motivation for that new kind of model is that fluid-solid interaction solutions are not sufficient to understand the enlargement and rupture risk of intracranial aneurysms. It is also necessary to include information about the mechanobiological responses by the cells to the computed hemodynamic loads. In that kind of models, traction-free boundary conditions at vascular model outlets are no longer realistic. Instead, impedance outlet boundary conditions are needed to yield physiologic pressure and flow waves in distensible multivessel models. Other modifications should also be considered to get an efficient coupling of fluid and solid domains. The authors propose that the coupled momentum method for fluid structure interaction (CMM-FSI), which incorporates equations for the vascular wall at a variational level as a boundary condition for the fluid domain, should be used instead of the standard arbitrary Lagrangian-Eulerian method. In that direction, Di Achille and Humphrey [150] presented the first approach toward large-scale computational fluid-solid-growth models of intracranial aneurysms, which are thought to be a reliable method to estimate evolution in time of the biomechanical properties of the wall and the remodeled vascular geometry. Three patient-specific models harboring intracranial aneurysms were reconstructed from CT angiography images, including a full Circle of Willis. Normalized flow waveforms were imposed at the inflows of those models for an assumed heart beat rate of 60 bpm. The Windkessel model was utilized to impose boundary conditions at the outlets of the models. Material stiffness was prescribed, corresponding to a uniform wall thickness of 0.36 mm. Newtonian rheology was assumed. In general, the average velocity at the core of the aneurysms was higher at the end of the systole if a rigid model was used. At the end of the diastole, differences were less pronounced. In one patient, maximum WSS was three times larger for the rigid wall model, but slightly higher for the compliant model at the end diastole. In another patient the WSS values at the end systole ranges between 25 and 30 Pa for the rigid wall model, while for the deformable wall those values ranged between 15 and 20 Pa.

## 9. Further Reading

During the last years, a number of review articles have addressed related topics. Taylor and Steinman [151] reviewed current techniques for image-based modeling of blood flow and vessel wall dynamics. Some years earlier, Steinman and Taylor [54] briefly discussed flow image and computational methods to study hemodynamics in large arteries. Steinman [152] also reviewed how advances in computational techniques are improving the understanding of the biomechanical behavior of the healthy and diseased cardiovascular system. In 2012, Jeong and Rhee [153] reviewed current commercial software and applications to cerebral aneurysm progress and treatment. Cebral and Raschi [154] reviewed works about suggested connections between risk factors of intracranial aneurysms. Sforza et al. [14] reviewed recent progress on the basic mechanisms of aneurysm formation and evolution, with a focus on the role of hemodynamic patterns. Dolan et al. [86] reviewed works focused on the effect of high wall shear stress and spatial gradients in vascular pathology. Taylor and Figueroa [155] reviewed methods to create anatomic and physiologic models, obtain properties, assign boundary conditions, and solve the equations governing blood flow and vessel wall dynamics. Augsburger et al. [156] reviewed current experimental methodologies and numerical approaches available for estimation of flow patterns, velocities, pressure, and their derived quantifications, such as wall shear stress and vorticity, by direct measurements or calculated through computation. Hoskins and Hardman [157] reviewed techniques for the estimation of wall stresses in arterial disease.

## 10. Discussion and Conclusions

Approximately twelve million people in the United States have cerebral aneurysms. Yearly, about 30,000 new patients with subarachnoid hemorrhage following rupture are reported. Given that only a very little portion of those lesions rupture, prophylactic interventions should be only for those patients who are more likely to rupture. Under this scenario, there is an increasing need to accurately determine the risk factors on an individual basis. The development of computational methods for the study of blood flow in patient-specific domains reconstructed from medical imaging has advanced the understanding of the mechanisms of interaction between the flow and the arterial wall, the initiation and development of cerebrovascular diseases, and the flow alterations generated by endovascular devices, which helps in evaluating treatment options. The validation of these methodologies has captivated the interest of neurosurgeons and interventional neuroradiologists who have seen a potential clinical application. Those experiments showed that numerical simulations performed over patient-specific domains and personalized boundary conditions are in agreement with blood flows measured in vivo with a variety of image modalities. Furthermore, those solutions show little or no dependence on most model assumptions and parameters. Different frameworks have been developed and utilized by some research teams to investigate possible associations between computed blood flows and cerebral aneurysm initiation, growth, and rupture, as well as intra-aneurysmal thrombus formation. While many questions remain unanswered, a large number of researchers worldwide are devoted to designing new approaches, algorithms, image modalities, and a wide variety of in vivo, ex vivo, and in silico experiments to answer them.
